# Supplementary material for: Healing of Skin Wounds in Rats Using Creams Based on Symphytum Officinale Extract
Source: Int J Mol Sci. 2024 Mar 7;25(6):3099. doi: 10.3390/ijms25063099 (PMC10970371; doi:10.3390/ijms25063099)
Supplement: Supplementary file 1 [file ijms-25-03099-s001.zip › ijms-2873350-supplementary.pdf]

Supplementary information

Healing of Skin Wounds in Rats Using Creams Based on Symphytum Officinale Extract

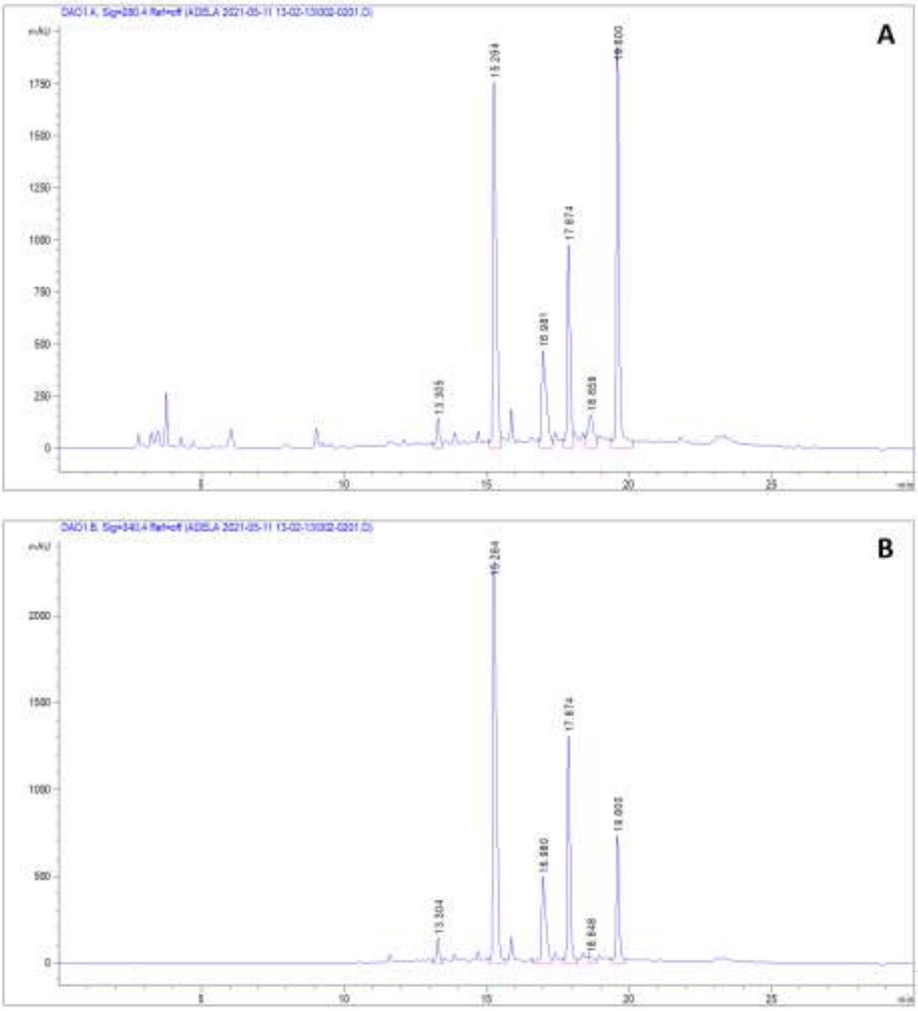

Figure S1 Comfey water extract chromatogram at 280nm (A) and at 340 nm (B)

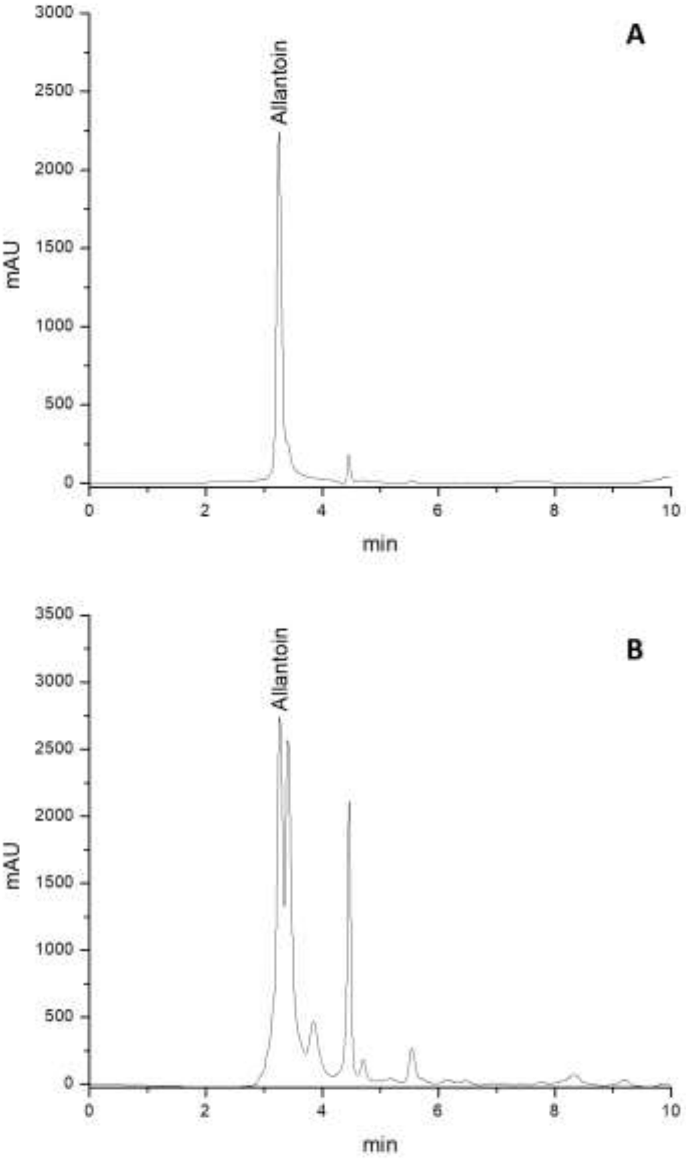

Figure S2 Chromatogram of Allantoin standard; Rt=3.25 min (A) and Chromatogram of Symphytum officinale extract; Rt=3.26 min (B)

Table S1. Histological analyses 8 and 14 days in terms of evaluating the SyOf regeneration efficiency

Days	Used cream	Histological modifications
8	Simple C	- the cutaneous defect is filled with granulation tissue that is covered at its surface by crust and on the marginal zones of the wound by surface epithelium.<br>- the fibers, basically collagen, are more prominent in this region as compared to the superficial zone of the granulation tissue.<br>- the granulation tissue's margins and the scar tissue's periphery are where the superficial epidermis is acanthotic, indicating the skin's reepithelialization.
8	SyOf 10%	- the cutaneous defect is filled with granulation tissue in the histological sections from the SyOf 10% group, essentially regenerating the entire dermis and hypodermis.

		- the inflammatory cells, primarily neutrophils and macrophages, are visible on the surface of the granulation tissue at the point where it connects to the crust, which is conspicuous in the center of the defect.<br><br>- the center region of the defect still contains crustal cell debris, but the defect's periphery exhibits skin reepithelization.
8	SyOf 20%	- acanthosis and cellular spongiosis are visible in the freshly formed epidermis, which completely covers the defect. Fibroblasts and a small amount of fibrocytes highly cellularize the granulation tissue in the dermis.<br><br>- the inflammatory infiltration is characterized by a dispersed population of neutrophils and macrophages, with multinucleated large cells from the macrophage lineage being seen in the marginal zone.
14	Simple C	- after 14 days, histological analysis of the cream control group's sections revealed that the surgical site had fully healed and the epidermis was continuous.<br><br>- Figure 11 A, B, E, and F show the many pilosebaceous units in the superficial and deep dermis.<br><br>-I n the section, the panniculus carnosus is visible (black star, Fig. 11 A, B).
14	10%	- the surgical incision was fully epithelialized in the histological sections from the SyOf 10% group after 14 days, and the pilosebaceous units partially regenerated, particularly at the defect's borders (thin arrow, Fig. 12 B, C, F, G).<br><br>- thick bundles of collagen fibers aligned parallel to the epidermis comprised the dense-fibrous connective tissue (scar tissue) that mostly replaced the superficial and deep dermis (Black stars, Fig. 12 C, G).<br><br>-in the deep dermis (thick arrow, Fig. 12 D), an amorphous, basophilic foreign substance is seen without a significant inflammatory response.
14	20%	- the surgical lesion was healed and covered by a regular epidermis in the histological sections from the SyOf 20% group after 14 days.<br><br>- there were few eosinophils, plasma cells, and a large number of macrophages in the deep dermis (black star, Fig. 13 D).<br><br>-within the cytoplasm of a small number of macrophages, there was an amorphous, basophilic foreign substance that was thought to be the test material (Fig. 13 D).
